# Discovery and Characterization of MaK: A Novel Knottin Antimicrobial Peptide from *Monochamus alternatus*

**DOI:** 10.3390/ijms242417565

**Published:** 2023-12-17

**Authors:** Xiaohong Han, Tong Zhou, Xinran Hu, Yukun Zhu, Zengzeng Shi, Shi Chen, Yunfei Liu, Xiaoqian Weng, Feiping Zhang, Songqing Wu

**Affiliations:** 1 College of Forestry, Fujian Agriculture and Forestry University, Fuzhou 350002, China; hxhdax@163.com (X.H.); 18208368769@163.com (X.H.); zhuyukun001@126.com (Y.Z.); fafuszz@163.com (Z.S.); 15633843711@163.com (S.C.); lyf522630@163.com (Y.L.); shakira1105@163.com (X.W.); 2 Key Laboratory of Integrated Pest Management in Ecological Forests, Fujian Province University, Fujian Agriculture and Forestry University, Fuzhou 350002, China; 3 Institute of Insect Sciences, College of Agriculture and Biotechnology, Zhejiang University, Hangzhou 310058, China; 15659555866@163.com; 4 Fujian Colleges and Universities Engineering Research Institute of Conservation and Utilization of Natural Bioresources, Fujian Agriculture and Forestry, Fuzhou 350002, China

**Keywords:** *Monochamus alternatus*, *Bursaphelenchus xylophilus*, knottin-type peptide, MaK, antimicrobial peptide, bioinformatics analysis, bioactivity

## Abstract

Knottin-type antimicrobial peptides possess exceptional attributes, such as high efficacy, low vulnerability to drug resistance, minimal toxicity, and precise targeting of drug sites. These peptides play a crucial role in the innate immunity of insects, offering protection against bacteria, fungi, and parasites. Knottins have garnered considerable interest as promising contenders for drug development due to their ability to bridge the gap between small molecules and protein-based biopharmaceuticals, effectively addressing the therapeutic limitations of both modalities. This work presents the isolation and identification of a novel antimicrobial peptide derived from *Monochamus alternatus*. The cDNA encodes a 56-amino acid knottin propeptide, while the mature peptide comprises only 34 amino acids. We have labeled this knottin peptide as MaK. Using chemically synthesized MaK, we evaluated its hemolytic activity, thermal stability, antibacterial properties, and efficacy against nematodes. The results of this study indicate that MaK is an exceptionally effective knottin-type peptide. It demonstrates low toxicity, superior stability, potent antibacterial activity, and the ability to suppress pine wood nematodes. Consequently, these findings suggest that MaK has potential use in developing innovative therapeutic agents to prevent and manage pine wilt disease.

## 1. Introduction

*Monochamus alternatus* plays a significant role as the primary vector for pine wilt disease (PWD), recognized as one of the most challenging and destructive forest diseases globally. PWD affects a wide range of hosts, employs diverse transmission pathways, and is characterized by its rapid reproductive capacity and high mortality rates among pine trees [1,2,3,4,5]. Using conventional chemical control methods for pest management is associated with high costs and a tendency for resistance development. Thus, investigating new bio-based compounds with nematicidal properties is very important [6,7,8]. The transmission of pine wood nematodes via *M. alternatus* involves a complex interaction that stimulates immune responses, resulting in elevated production of natural compounds [2]. A promising approach for controlling PWD entails investigating the naturally occurring compounds created by *M. alternatus* to discover new potential nematocidal agents. The first stage of pest management involves studying and identifying suitable natural compounds obtained from the body of *M. alternatus*.

Insects comprise the most extensive and diverse species in the animal kingdom, equipped with various self-defense mechanisms such as physical barriers, innate cellular immunity, and humoral immune responses [9,10]. A crucial immune defense mechanism in insects involves clearing pathogens by producing antimicrobial peptides through the Imd signaling pathway [11,12]. Insect antimicrobial peptides (AMPs) are a class of small-molecule cationic peptides induced within the insect body and are members of the antimicrobial peptide family with shared functions. These peptides possess diverse properties, including antibacterial, antifungal, antiparasitic, antitumor, and antiviral activities. Moreover, they effectively mitigate the development of resistance [13,14,15,16,17]. Cecropin pxCECA1 mainly demonstrates activity against methicillin-resistant staphylococcus aureus (MRSA) [18]. On the other hand, thaumatin exhibits broad-spectrum antimicrobial properties and is effective against fungi and bacteria [19]. Furthermore, cecropin A is notable for its antiviral activity against HIV, and andropin shows efficacy as an antiparasitic agent [20,21]. However, due to the low stability of antimicrobial peptides in plasma, they are rapidly cleared by the liver and kidneys, making high stability crucial.

One distinct form of antimicrobial peptides is the knottin-type peptides, known for their unique structure. Knottins can be found in plants, animals, and insects, serving various functions. Generally composed of 30 to 50 amino acids, these peptides are identified by their core consisting of antiparallel β-strands. This structure is further reinforced by at least three disulfide bonds, resulting in a macrocyclic configuration through pairings like C1-C4, C2-C5, and C3-C6 [22,23,24]. Peptides with this structural configuration exhibit exceptional chemical, thermal, and proteolytic stability, presenting promising prospects for drug development and diagnostic applications [25,26,27]. Notably, their remarkable stability opens novel opportunities for oral drug delivery, unlike other protein therapies, which are typically ill-suited for oral administration due to the distinct conditions of the gastrointestinal tract. The naturally occurring cysteine knottin peptide Linzess1, which has received FDA approval, is effectively delivered orally to address chronic constipation and irritable bowel syndrome [28]. In addition, research has aimed to develop innovative amino acid sequences that can target tumor-associated biomarkers and expand the range of applications for knottins in cancer. This is particularly significant as they promise intracellular targeting [29,30,31]. Moreover, the compact size of knottins offers a distinct advantage by enabling their expression through microbial recombinant protein production or easy chemical synthesis [32].

Utilizing knottin peptides, naturally synthesized by *M. alternatus*, as targeted drugs for combatting the pine wood nematodes offers a promising solution to the resistance issue while also serving as an environmentally friendly plant protector [33]. In this study, we have isolated a novel knottin peptide named MaK from *M. alternatus* and conducted comprehensive structural and functional analyses, confirming its antibacterial and nematocidal properties. These findings lay a solid foundation for using MaK as an engineered knottin to prevent and control pine wood nematodes (PWD).

## 2. Results

### 2.1. The Structure of Knottin-Type Peptide MaK

**Figure 1:**
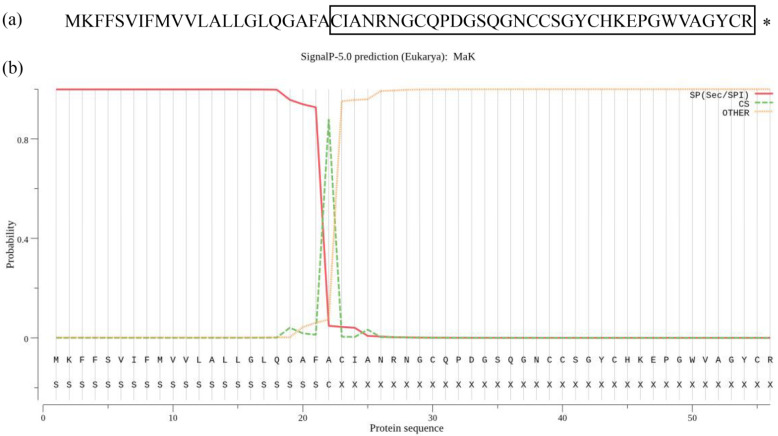
The amino acid sequence of *M. alternatus* knottin-type peptide and prediction results in MaK signaling peptide. (**a**) The amino acid sequence information of MaK, with boxed regions representing the mature peptide segment (consisting of 34 amino acids) and the shop codon with “*”. (**b**) Prediction results for MaK’s signal peptide and propeptide cleavage sites. S-score (signal peptide score) is utilized to distinguish the respective location as a signal peptide region. C-score (raw cleavage site score) is used to determine the cleavage site, with the highest peak indicating the first amino acid residue following the cleavage site (the first amino acid residue of the mature protein).

We have identified a sequence highly homologous to a knottin-type antimicrobial peptide by analyzing the transcriptome data of *M. alternatus*. Our examination has unveiled that this sequence codes for a knottin peptide comprising 56 amino acid residues (Figure 1a). Figure 1b illustrates the precursor composition of this peptide, which consists of a 22-amino acid signal peptide and a 34-amino acid mature peptide known as MaK. The calculated molecular weight for the peptide is 6020.04, with a theoretical isoelectric point of 8.29. Assessing the instability index, the MaK protein demonstrates a value of 30.82, indicating its stability.

### 2.2. Structural Analysis and Homology Alignment of Knottin-Type Peptide MaK

**Figure 2:**
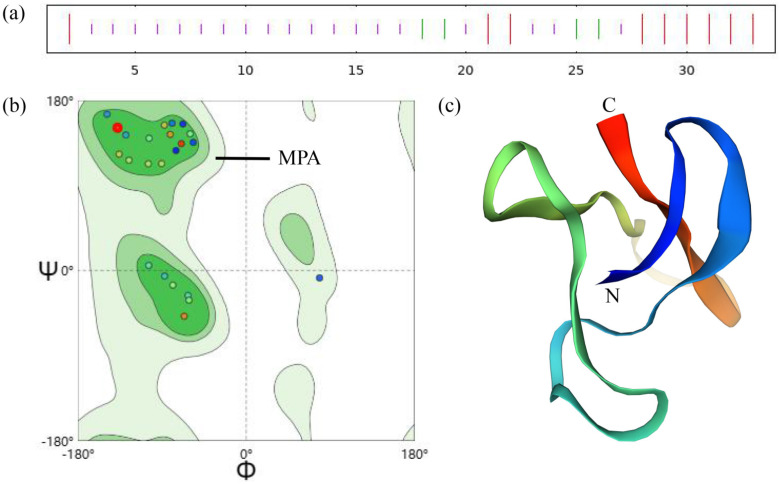
Secondary and tertiary structure prediction results for *M. alternatus*. (**a**) The secondary structure of the MaK protein (red: extended strand, green: β-turn, purple: random coil). (**b**) The general Ramachandran plot for proteins illustrates the dihedral angles of the α-carbon, where ϕ signifies the rotation angle of the C-N bond to the left of the α-carbon within a peptide unit, and ψ represents the rotation angle of the C-C bond to the right of the α-carbon. MPA: maximum permissible area. (**c**) The tertiary structure of the MaK protein. N/C: denoting the N/C termini of the protein.

The investigation of the secondary structure prediction for the MaK protein indicates that 11 amino acid residues adopt an extended strand conformation, 4 amino acid residues form a beta-turn, and 19 amino acid residues are in a random coil configuration. These constitute 32.35%, 11.76%, and 55.88% of the secondary structure composition (Figure 2a). The results of homology modeling for the three-dimensional structure of the MaK protein demonstrate that over 90% of the amino acid residues are situated within the permitted and highly favored regions, with no residues observed in disallowed regions. These findings suggest that the proposed model adheres to stereochemistry principles (Figure 2b). The tertiary structure of the knottin peptide MaK exhibits similar characteristics to other knottin peptides. These peptides form 3pairs of disulfide bonds to create a stable cyclic structure (Figure 2c).

**Figure 3:**
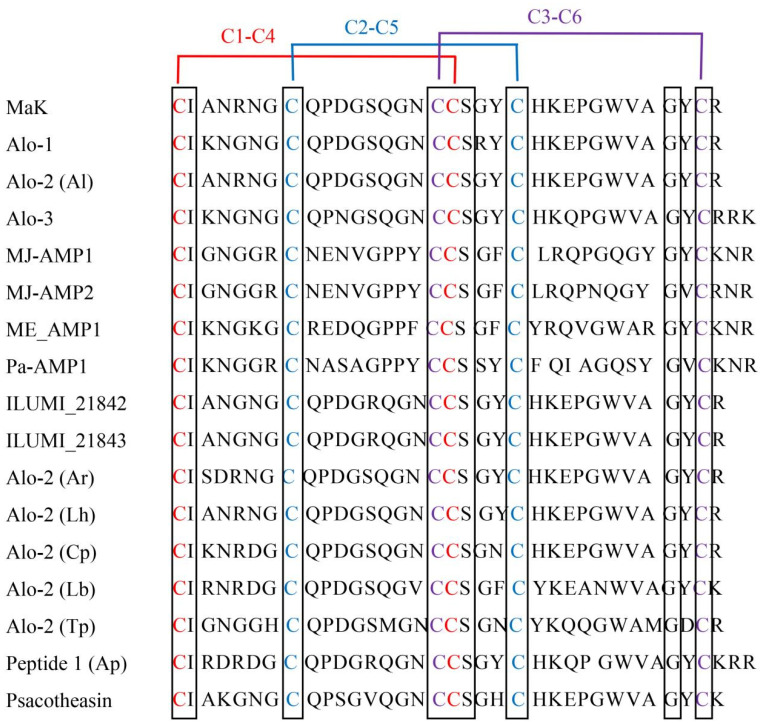
Sequence alignment of MaK with other knottin-type peptides.

Knottin peptides exhibit remarkable characteristic conservation. Our homology analysis with other knottin peptides revealed a consistent pattern: these peptides typically consist of 30 to 50 amino acids in length. Moreover, sequence alignment consistently demonstrates similar structural features among these peptides. All these sequences contain six stably expressed cysteine residues, forming the following three intramolecular disulfide bonds in a paired pattern to ensure the stable structure of these peptides (Figure 3): C1-C4, C2-C5, and C3-C6.

### 2.3. Peptide Synthesis

The knottin peptide MaK was purified by HPLC with a peptide purity > 95%, as shown in Figure S1.

### 2.4. Antimicrobial Activity Testing of Knottin-Type Peptide MaK

**Figure 4:**
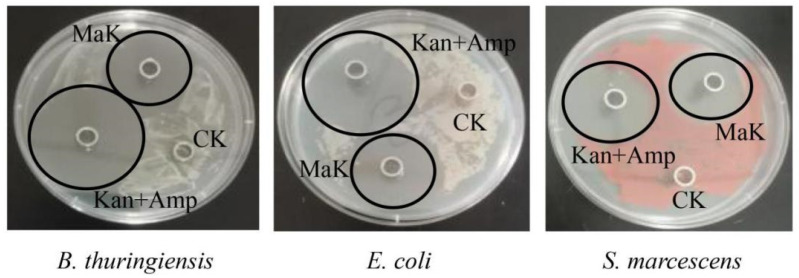
The antibacterial activity of knottin-type peptide MaK. The MIC was determined by using the 96-well plate method, and the presence of inhibition zones was assessed by utilizing the agar diffusion method.

The synthesized knottin peptide MaK was used to assess its antibacterial activity against Gram-positive and Gram-negative bacteria. The results presented in Table 1 indicate that all tested strains are susceptible to MaK. The MIC of MaK against *Bacillus thuringiensis* and *Escherichia coli* was 36.29 µg/mL, and against *Serratia marcescens* was 72.58 µg/mL, indicating significant antibacterial activity. Moreover, in the inhibition assay, the peptide exhibited distinct inhibitory zones against all three bacterial strains in the inhibition assay (Figure 4). After MaK processing, the inhibition zone diameters for *B. thuringiensis* was 2.087 ± 0.13 cm, for *E. coli* was 2.053 ± 0.08 cm, and for *S. marcescens* was 1.58 ± 0.072 cm.

### 2.5. Nematocidal Activity Testing of Knottin-Type Peptide MaK

**Figure 5:**
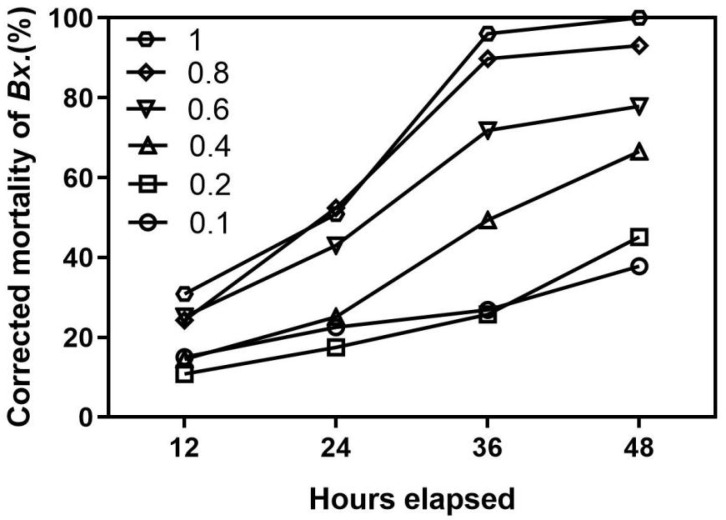
Nematocidal activity of knottin-type peptide MaK. Conducting a pinewood nematode bioassay using a 96-well plate method. 0.1, 0.2, 0.4, 0.6, 0.8, 1.0 correspond to MaK concentrations of 0.1, 0.2, 0.4, 0.6, 0.8, and 1.0 mg/mL, respectively.

The findings shown in Figure 5 make it apparent that MaK exhibits a potent lethal effect on pine wood nematodes. There is a clear dose–response relationship, where an increase in MaK concentration leads to a corresponding increase in lethality against these nematodes. The mortality rate of pine wood nematodes notably increases when the concentration reaches 1 mg/mL. Following a 48 h treatment period, the mortality rate of pine wood nematodes rises to 100%, with an LC_50_ = 0.362 mg/mL.

### 2.6. Hemolytic Activity Assay of Knottin-Type Peptide MaK

**Figure 6:**
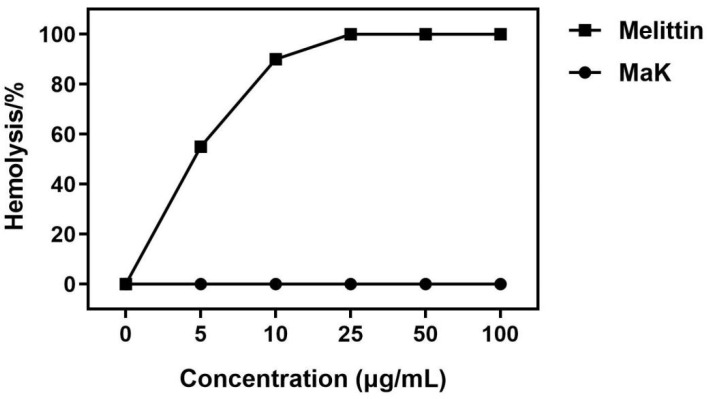
Hemolytic activity of knottin-type peptide MaK.

We investigated to assess the hemolytic activity of MaK on red blood cells derived from mice. In contrast to melittin, which has been previously demonstrated to possess significant hemolytic activity, our experiments revealed that MaK does not exhibit any hemolytic activity on mouse red blood cells, as illustrated in Figure 6. These results provide strong evidence indicating that the knottin peptide, MaK, does not induce cytotoxic effects on mammalian red blood cells.

### 2.7. Thermal Stability Assessment of Knottin-Type Peptide MaK

**Figure 7:**
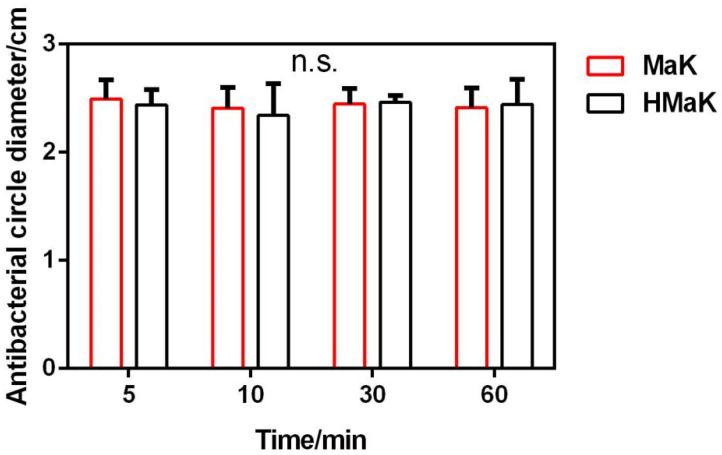
Thermal stability of knottin-type peptide MaK. HMaK represents heating treated knottin-type peptide MaK. There is no significance with “n.s.” (*p* > 0.05).

The thermal stability of MaK was evaluated by subjecting it to treatment at 100 °C for different durations, namely 5, 10, 30, and 60 min. We measured the inhibition zone sizes and conducted a statistical analysis. Figure 7 illustrates that there was no significant difference in inhibition zone sizes after the various heat treatments, and the antibacterial activity of MaK remains unaffected. Hence, we can infer that the efficacy of MaK is not compromised by varying heat treatment durations, indicating its impressive heat resistance and structural stability.

## 3. Discussion

The exploration and utilization of insect resources have long been a significant interest. Identifying novel antimicrobial peptides holds great promise for addressing pathogens, treating diseases, cancer therapy, and protecting crops [34,35,36]. In this study, we utilized transcriptomic data from *M. alternatus* larvae to successfully identify and characterize a previously unidentified knottin peptide named MaK. Through comprehensive analyses, including investigations into the gene structure, physicochemical properties, spatial structure, and homology, we have established a solid foundation for further understanding the functional aspects of this gene. The identification results suggest that MaK is a stable protein and demonstrates considerable antibacterial and nematocidal properties. However, further investigation is required to explore the precise underlying mechanisms of its actions. The diversity of insect antimicrobial peptides is closely associated with the environmental and survival threats each insect faces during evolution. As insects encounter a wider range of pathogens, the number of antimicrobial peptide types produced increases. Antimicrobial peptides perform immunomodulatory functions as crucial constituents of the host immune system [37]. In the transcriptome data of *M. alternatus*, the expression level of these antimicrobial peptides are remarkably high, indicating significant changes in the physiological activities of the larvae during this period, triggering an immune response and leading to an abundant expression of these antimicrobial peptides, warranting further investigation.

There is an urgent need to develop nematocides for managing pine wood nematode disease. Nevertheless, the utilization of chemical agents is greatly restricted because of their notable adverse consequences on biological safety and the environment, both in direct and indirect ways [38,39]. To decrease dependence on chemical agents, exploring the use of naturally occurring compounds produced by insects holds the potential for creating innovative nematocides [40]. Studies have indicated that intrinsic factors, such as physicochemical characteristics and spatial structure, predominantly influence the biological activity of antimicrobial peptides. Knottin peptides are known for their remarkable stability and sequence flexibility, which makes them highly suitable for targeted in vivo applications [41,42,43,44]. Their exceptional flexibility allows for introducing new functions via chemical synthesis or modifications, allowing them to adapt well to various usage environments. Consequently, in addition to the direct utilization of natural knottin peptides, the construction of engineered knottin peptides tailored to specific application scenarios can achieve even more precise and effective results. Obtaining antimicrobial peptides from insect sources is challenging, with chemical synthesis and genetic engineering being the primary methods for their extraction. Chemical synthesis is favorable for smaller-sized insect antimicrobial peptides, as it not only reduces costs but also yields peptides with high purity. Consequently, the chemical synthesis of antimicrobial peptides has become a critical approach for studying the structure of small-molecule insect antimicrobial peptides and rapidly obtaining peptide products.

Antibiotics and melittin peptides are known for their potent broad-spectrum antibacterial activity but often lack selectivity towards normal cells. In recent years, antimicrobial peptides have garnered attention as substitute candidates for new drugs in the clinical field, aiming to overcome microorganisms without easily developing drug resistance [45,46,47]. This study aims to discover novel peptides with vigorous antibacterial and nematicidal activities while demonstrating minimal or no cytotoxicity and high stability. This holds tremendous potential for developing new antimicrobial drugs, an innovative approach, and bioactive compounds to prevent and control pine wood nematode diseases. Knottin-type peptides incorporating the ICK motif demonstrate exceptional stability against heat denaturation and protein degradation. These peptides have become valuable assets in disease treatment due to their robustness [22,26,32]. Extensive investigation has revealed that this peptide class effectively displays antimicrobial, antifungal, and antiparasitic properties [32,33]. In our experiments, applying the peptide directly to pine wood nematodes considerably increased in mortality rates. Additionally, it is crucial to examine the interplay between the peptide *M. alternatus* and *B. xylophilus* to optimize its effectiveness in inducing pine wood nematode mortality.

## 4. Materials and Methods

### 4.1. Transcriptomic Data Filtering and Sequence Analysis

Antimicrobial peptides were screened in the larvae of *M. alternatus*, utilizing the transcriptomic data obtained from a previous study (accession number SRP070969) [48]. Sequence alignment and analysis were conducted using the NCBI BLAST program (http://www.ncbi.nlm.nih.gov/BLAST) (accessed on 16 March 2022). Signal peptide analysis was conducted using the ProP 1.0 Server (http://www.cbs.dtu.dk/services/ProP/) (accessed on 16 March 2022), while we predicted the physicochemical properties using the ProtParam tool (https://web.expasy.org/protparam/) (accessed on 16 March 2022).

### 4.2. Structural Analysis and Homology Comparison of Knottin-Type Peptide MaK

The secondary structure of MaK was predicted using the SOPMA tool (https://npsa-prabi.ibcp.fr/cgi-bin/npsa_automat.pl?page=npsa_sopma.html) (accessed on 16 March 2022). Following this, the three-dimensional structure of the mature peptide MaK was predicted using the homology modeling method available on SWISS-MODEL (https://www.swissmodel.expasy.org/) (accessed on 16 March 2022). To analyze the alignment between the amino acid sequence of MaK and 16 other knottin peptides, we utilized Clustal Omega (https://www.ebi.ac.uk/Tools/msa/clustalo/) (accessed on 16 March 2022) for this purpose. The results of the alignment are shown in Table S1.

### 4.3. Peptide Synthesis

The synthesis of the MaK knottin peptide was outsourced to Wuhan Dangang Biotechnology Co., Ltd. (Wuhan, CN, USA), who utilized the solid-phase chemical synthesis method. The peptide was synthesized using the standard Fmoc method and purified by HPLC, confirming the relative molecular weight via mass spectrometry. The compound was stored at −20 °C for future use.

Chromatographic conditions: The chromatographic column was Kromasil-C_18_ (250 mm × 4.6 mm, 5 μm); the mobile phase consisted of 0.1% TFA in acetonitrile (A) and 0.1% TFA in water (B) with gradient elution (0 to 25 min, 0% A–15% A; 25.0 to 25.1 min, 40% A–100% A; 25.1 to 30.0 min, 100% A).

Mass spectrometry conditions: Electrospray ionization (ESI) was utilized in a positive ion mode with a spray voltage of 4500 V. The scan range was set to *m/z* 400–2000. The interface temperature was maintained at 350 °C, while the desolvation line (DL) temperature was set to 250 °C. The nebulizer gas flow rate was 1.5 min^−1^, and the heat block temperature was 400 °C.

### 4.4. Measurement of the Antimicrobial Activity of Knottin-Type Peptide MaK

The antimicrobial activity of the MaK knottin peptide was assessed using the method of determining the minimal inhibitory concentration (MIC) [33]. *B. thuringiensis*, a Gram-positive bacterium, as well as *E. coli* and *S. marcescens*, both Gram-negative bacteria, were cultivated until they reached the logarithmic growth phase and then suspended at a concentration of 10^8^ CFU/mL. Ninety microliters of each bacterial suspension were added to individual wells of a 96-well cell culture plate. A 10 µL solution of the MaK knottin peptide was added, gradually decreasing in concentration with each subsequent addition by a factor of two. During the experiments, sterile water was utilized as the negative control, while ampicillin and kanamycin were employed as the positive controls. Each condition was replicated three times. The absorbance values were measured before and after cultivation to ascertain the minimum peptide concentration necessary to inhibit microbial growth.

Concurrently, a disk diffusion assay was conducted using the aforementioned bacterial strains. The frozen strains were activated, and 100 µL of cultured bacterial suspension was spread on an LB solid medium. Subsequently, three sterile Oxford cups were placed on each Petri dish, each cup containing 100 µL of ampicillin and kanamycin solution, MaK knottin peptide solution, and sterile water, respectively. The petri dishes were then incubated in a culture chamber with three replicates. After 12 h of incubation, inhibition zones were examined.

### 4.5. The Nematocidal Action of Knottin-Type Peptide MaK

This study utilized a laboratory strain of pine wood nematodes (PWN), and the inoculation process involved introducing PWN onto potato dextrose agar (PDA) medium. Following an incubation period of 7–10 d, a suspension of PWN was added to the culture dish containing PDA. The dish was then placed within a dark incubator set at a temperature of 28 °C, promoting the cultivation of PWN. Subsequently, the separation of PWN was achieved using the Baermann funnel method. The PWN was observed and quantified using a dissecting microscope. The MaK knottin peptide was diluted with sterile water to establish concentration gradients to achieve concentrations of 0.1, 0.2, 0.4, 0.6, 0.8, and 1 mg/mL. Subsequently, 10 µL of the diluted MaK knottin peptide solution was added to each well of a 96-well plate, followed by the addition of 90 µL of nematode suspension (approximately 30 nematodes per well). For the blank control, sterile water was employed, and we ensured that three replicates are set up. The samples were cultivated in a 28 °C incubator, and we carefully monitored the activity of pine wood nematodes. The number of deceased nematodes was recorded using a dissecting microscope at 12 h intervals. Finally, the adjusted mortality rate was calculated.

### 4.6. Hemolytic Activity Assay of Knottin-Type Peptide MaK

To evaluate the hemolytic activity of the MaK knottin peptide, its impact on hemoglobin obtained from a suspension of mouse red blood cells was measured [49]. After mixing the peptide dilutions with the mouse red blood cells at different concentrations, the mixture was incubated at 37 °C for 1 h. Subsequently, the incubated red blood cells were centrifuged at 4 °C (1000 r/min, 10 min). The absorbance was measured at a wavelength of 414 nm, with the mouse red blood cells without the peptide as the negative control and the mouse red blood cells treated with 0.1% Triton X-100 as the positive control. The same method was employed using Melittin to treat the mouse red blood cells. The hemolysis rate can be calculated using the following formula: hemolysis rate (%) = (Abs414_nm_ in the pepide solution − Abs414_nm_ in saline)/(Abs414_nm_ in 0.1% Triton X-100 − Abs414_nm_ in saline) × 100%.

### 4.7. Thermal Stability Testing of Knottin-Type Peptide MaK

A solution of the MaK knottin peptide at a concentration five times greater than the MIC was prepared. Subsequently, the solution to heat treatment by immersing it in boiling water at 100 °C for 5, 10, 30, and 60 min was esxposed, with the untreated MaK knottin peptide solution as the control. Overnight cultured *E. coli* was diluted and spread onto LB solid medium, followed by placing of Oxford cups containing the MaK solution treated at various temperatures (HMaK) and the untreated MaK solution. The petri dishes were then incubated at 37 °C for 12 h in a constant temperature incubator, and subsequently, the sizes of the inhibition zones against *E. coli* were measured.

### 4.8. Statistical Analysis

The data from this experimental study were measured in multiple parallel replications. Statistical analysis was performed using SPSS 22.0 software, employing *t*-tests or one-way analysis of variance (ANOVA), with *p* < 0.05 indicating statistical significance. Graphs were generated using the Graphpad Prism 8 software.

## 5. Conclusions

This study adds to the knowledge of antimicrobial peptides in *M. alternatus*. In particular, we identified a short yet stable antimicrobial peptide. Our investigation focuses on understanding the structure and function of MaK, which is a knottin peptide derived from *M. alternatus.* MaK is a natural knottin protein exclusively found in the larvae of *M. alternatus* and shows a high level of expression during this developmental stage. With its compact 34-amino acid sequence, MaK exhibits a low toxicity profile, exceptional stability, remarkable antibacterial activity, and potent inhibitory effects on pine wood nematodes. These findings have significant implications and provide a solid foundation for investigating new natural compounds that could be used in preventing and managing pine wood nematode disease.

## Figures and Tables

**Table 1:** MIC value determination of knottin-type peptide MaK.

Bacterial Strains		MIC (µg/mL)	
		MaK	Kan + Amp
Gram-positive	*B. thuringiensis*	36.29	<36.29
Gram-negative	*E. coli*	36.29	<36.29
	*S. marcescens*	72.58	<36.29

## Data Availability

The data are not publicly available due to commercial value. Interested parties can email corresponding authors.

## Supplementary Materials

The supporting information can be downloaded at: https://www.mdpi.com/article/10.3390/ijms242417565/s1.
