# Supplementary material for: Discovery and Characterization of MaK: A Novel Knottin Antimicrobial Peptide from Monochamus alternatus
Source: Int J Mol Sci. 2023 Dec 17;24(24):17565. doi: 10.3390/ijms242417565 (PMC10743862; doi:10.3390/ijms242417565)
Supplement: Supplementary file 1 [file ijms-24-17565-s001.zip › ijms-2762423-supplementary.pdf]

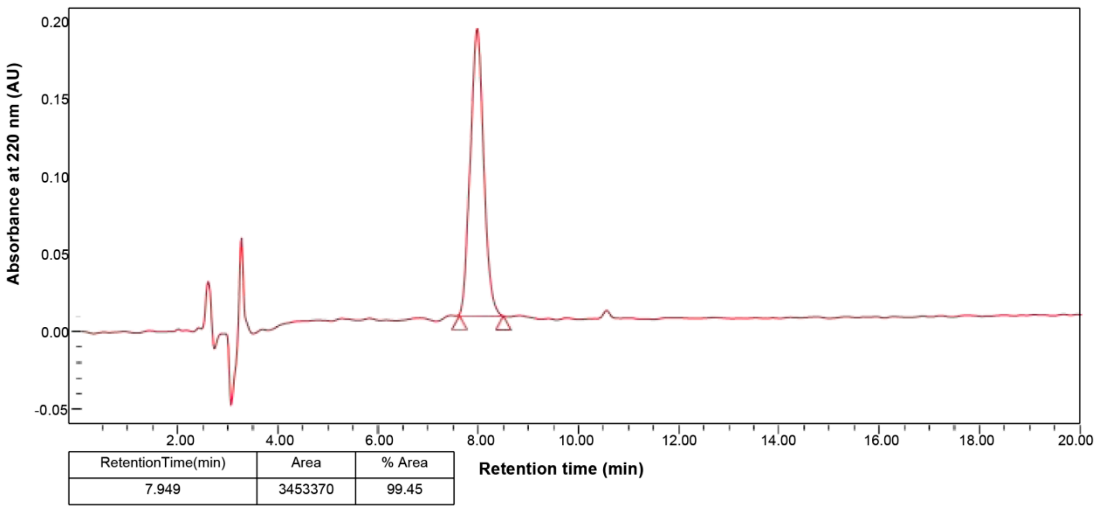

Figure S1. Purity of synthesized MaK detected by HPLC

Table S1. Species for homology analysis

Knottin peptides	Species	GenBank Accession Number/DOI
Alo-1	*Acrocinus longimanus*	P83651.1
Alo-2 (Al)	*A. longimanus*	P83652.1
Alo-3	*A. longimanus*	P83653.1
MJ-AMP1	*Mirabilis jalapa*	P25403.4
MJ-AMP2	*M. jalapa*	P25404.2
ME_AMP1	*Mesembryanthemum crystallinum*	O81338.1
Pa-AMP1	*Phytolacca americana*	P81418.1
ILUMI_21842	*Ignelater luminosus*	KAF2884323.1
ILUMI_21843	*I. luminosus*	KAF2884324.1
Alo-2 (Ar)	*Athalia rosae*	XP_048514267.1
Alo-2 (Lh)	*Leptopilina heterotoma*	XP_043469554.1
Alo-2 (Cp)	*Conogethes punctiferalis*	QRV07324.1
Alo-2 (Lb)	*Leptopilina boulardi*	XP_051170841.1
Alo-2 (Tp)	*Trichogramma pretiosum*	XP_014233229.1
Peptide 1 (Ap)	*Agrilus planipennis*	XP_018321118.1
Psacotheasin	*Psacothea hilaris*	DOI: 10.4014/jmb.1002.02003
